# An Attributional Analysis of Stigma Associated with Sexually Transmitted Diseases and Its Relationship with Communication Efficacy

**DOI:** 10.5539/gjhs.v4n4p15

**Published:** 2012-05-21

**Authors:** Jina H. Yoo, Suahn Jang

**Affiliations:** 1 University of Missouri - St. Louis, USA

**Keywords:** stigma, STD, communication efficacy, attribution of responsibility, Merck’s gardasil advertisement

## Abstract

People typically attribute more responsibility to those individuals who are infected with sexually transmitted diseases (STD) than other diseases. This study tested how different routes (i.e., sexually transmitted or foodborne) of transmission have an impact on individuals’ general perception on stigma/shame and the attributions of responsibility, when controlling for symptoms/conditions of the hypothetical virus infection. Two hundreds and ninety eight college students were recruited for the study. As predicted, people who were attributed with control over contracting the virus (i.e., sexually transmitted route) were likely to be assigned a greater level of personal responsibility and were more likely to receive blame from others than people who were attributed relatively less control over contracting the virus (i.e., foodborne). The relationship between the attribution of responsibility and communication efficacy was also assessed. The results supported our prediction that there was a significant association between the attribution of responsibility and communication efficacy, in that the perceived controllability of the situation, perceived responsibility for the situation, and blame were all significantly correlated with communication efficacy in a negative direction. Practical applications by evaluating the effectiveness of the actual Merck’s Gardasil advertisement were discussed that the Gardasil advertisement appears to reduce the perceived shame and stigma associated with the sexually transmitted nature of the virus by not revealing the true nature of the virus upfront.

## 1. Introduction

STDs are problematic not only because they cause serious physical disabilities but also that they are closely associated with stigma (Smith, 2007a). People who have STDs are often stigmatized because they are viewed as having excessive sexual activities or sexual immortality (Lichtenstein, ook, & Sharma, 2005). The stigma associated with STDs usually acts as a potential barrier for patients in receiving an appropriate diagnosis and timely treatment, due to patients’ desire for secrecy (Cunningham, Tschann, Gurvey, Fortenberry, & Ellen, 2002). Considering the fact that stigma can function as a great barrier for health promotion and treatment, little research has been done in the field of health communication in order to understand how this perceived stigma has an impact on one’s communication behaviors. In addition, comparatively little attention has been given to the stigma associated with STDs that are not HIV/AIDS (Yankauer, 1994).

The purpose of this study is twofold. First, we attempted to assess individuals’ general attitude about STDs. Specifically, this study examined how different routes of transmission has an impact on individuals’ general perceptions on stigma and its attributions of responsibility when controlling for symptoms/conditions of the disease. Second, this study tried to explain how attributions of responsibility predicted individual’s communication efficacy in terms of disclosing about the disease. Especially, the present study proposed a slight modification of Weiner’s decision-stage model (Weiner, 1995) by testing the path model of controllability, responsibility, and blame, to communication behavior.

### 1.1 Stigma and STDs

Stigma is a social construct that is shared by members of a society, which has an impact on personal and group interactions. Fortenberry et al. (2002) defined stigma as “an attribute or label that sets a person apart from others and links the characteristics (p. 1-2).” Stigmatized individuals are discounted and discredited by others; as a result, they are more likely to lose their social status (Goffman, 1963). Not surprisingly, stigma has been demonstrated to have associations with stereotyping, prejudice, and discrimination.

It has been recognized by medical sociologists that various types of illnesses are associated with stigma. For instance, it has been frequently reported that individuals with HIV/AIDS have suffered from extreme stereotyping, associated with homosexuality or IV drug use (Green, 1995). In terms of prejudice and discrimination, evidence has demonstrated that individuals with HIV/AIDS have been denied health care services and employment (Herek, Capitanio, & Widaman, 2002). Although it has been not as widely studied as HIV/AIDS, individuals with a STD feel strain in the way they perceive themselves. Oftentimes, they suffer from low self-esteem by viewing themselves as dirty, promiscuous, or disgusting as a result of being diagnosed with an STD (J. Lee & Craft, 2002). Holzemer and colleagues (2009) investigated the quality of life in people living with HIV infection, and found that stigma increases the stress associated with the disease by contributing to psychological and social burden; consequently, it affects quality of life and physical well-being as a whole. Therefore, it is important to investigate the reason why STDs are frequently associated with stigma and how people in general perceive individuals with STDs.

According to Smith (2007b), health issues are typically presented in two formats: challenge and stigma formats. Smith indicated that the diseases presented in a challenge format typically have chronic and noninfectious conditions (e.g., cancer, heart disease), while the diseases described in a stigma format are infectious (e.g., AIDS, STDs, tuberculosis). Among those diseases, STDs, especially, appeared to be more stigmatized than other diseases (Smith, 2007a). Why is it the case? Many have argued that perceived responsibility plays a crucial role in forming stigma (Corrigan, Markowitz, Watson, Rowan, & Kubiak, 2003; Mak et al., 2006; Mantler, Schellenberg, & Page, 2003). Research on stigma often utilizes attribution theory in that individuals’ interpretations of the cause of the disease have a significant influence on their emotional and behavioral responses towards patients who have the disease (Weiner, 1993).

Stigma is intensified when the individuals who are infected are viewed as holding responsibility for the condition. When people make attributions of responsibility, they examine whether the disease carrier had control over the situation or not. When the public observe that infection of the disease was controllable by the individuals, in that, they knew about the potential outcomes of their own actions, then the public is more likely to hold the infected individuals responsible for their own illness (Corrigan et al., 2003). Most of the time, STDs are perceived to be the responsibility of the infected, since STDs are generally viewed as the result of engaging in voluntary sexual activity, usually perceived to be immoral (Lichtenstein et al., 2005). Based on this notion, the public seem to perceive that people with STDs had control over the situation. Compared to STDs, cancer is judged to be uncontrollable, in that people believe “it could happen to anyone.” Thus, the public tend not to hold cancer patients responsible; consequently, cancer patients tend not to be as severely stigmatized as the people living with STDs in society.

Responsibility and a perception of choice are closely related. The general public appears hold heavier responsibility to those who choose immoral beliefs or behaviors than those of who has involuntary ones. Consequently, when people perceive that infection of the disease could have been controllable by the individuals, they are more likely to blame and stigmatize the infected than those of who are infected inadvertently (Corrigan et al., 2003). In this sense, individuals who are infected with STDs are typically viewed as voluntarily choosing to engage in such immoral sexual acts (i.e., having multiple sexual partners, having sex with disreputable people, having one night stands, etc.). It makes it difficult for the infected to be free from blame in the public eye, consequently, they suffer from heavy stigmatization. Their study resonated with Smith’s (2007a) reflection on stigma that people are concerned not only about being infected with a contagious disease but also with their moral standing being damaged.

The stigma is closely connected to shame, which is defined as an intense negative emotion that emerges from an individual experiencing failure to meet his or her or other people’s standards, feeling personally responsible for that failure and believing that failure to reflect an inadequate self (Cunningham et al., 2002). Associated STDs with shame, along with stigma, are considered to be important barriers to receiving appropriate diagnostic and treatment services (Fortenberry et al., 2002).

According to Waller, Marlow, and Wardle (2007), women who knew that Human Papillomavirus (HPV) is a STD experienced higher levels of stigma, shame, and anxiety if they tested positive for the virus compared to women who were not aware of its transmission route. Most of the general public still remains unaware of the HPV and the sexually transmitted nature of the virus; consequently, the perceived stigma associated with HPV is relatively low compared to other STDs (e.g., HIV, gonorrhea, Chlamydia, etc.). Ironically, some researchers and clinicians have grown concerned that as there is greater public awareness of the HPV and its route of transmission, the greater perceived stigma will be established among people, which can act as a barrier in screening (Waller et al., 2007). In fact, the fear of STD-related stigma and shame associated with STDs are frequently reported as the main reasons for delays in screening or care-seeking (Cunningham et al., 2002; Fortenberry et al., 2002).

Accordingly, the pharmaceutical company, Merck, hid the fact that HPV is a sexually transmitted virus in their ads for the HPV vaccine Gardasil, despite the American Cancer Society’s recommendation that health practitioners should educate people that HPV is a STD (Anhang, Goodman, & Goldie, 2004). For instance, in TV commercials, on the official website (http://www.gardasil.com), and in printed brochures for Gardasil, the only information regarding HPV tend to emphasize its 1) prevalence (e.g., “4 out of 5 women will contract HPV. Will you?”, “It is estimated that 74% HPV occur in 15- to 24- year old.” etc.), and 2) involvement in the development of cervical cancer (e.g., “You could become 1 less life affected by cervical cancer.” or “Cervical cancer is caused by certain types of a common virus – HPV.”). The fact of the sexually transmitted nature of HPV is either not stated or is found in a small font on the back of the brochure. Perhaps it is a strategic move because concealing that HPV is a type of STD may have permitted people to talk freely about the disease without being afraid of becoming stigmatized by other people.

The current study proposed to examine if the phrase “sexually transmitted infection” has a significant effect on forming stigma. This study tested how different routes (i.e., sexually transmitted or foodborne) of transmission has an impact on individuals’ general perception on stigma/shame and the attributions of responsibility (controllability, responsibility, and blame), when controlling for symptoms/conditions of the hypothetical virus infection. Also, this study added one more condition, which is similar to Merck’s Gardasil advertisement. This condition contained the information that this hypothetical infection can develop into cancer, but the information regarding the route of transmission would not be revealed to participants. To this end, the first hypothesis stated:

H1: Among the following three conditions [(a) sexually transmitted route, (b) foodborne route, (c) no indication of transmission route, but its possible development for cancer], the sexually transmitted route will have the highest score for the perceived (1) controllability, (2) responsibility, (3) blame, (4) stigma, and (5) shame.

In addition, this study examined the impact of the word “cancer” on individuals’ perceived health threat. According to Witte’s (1994) Extended Parallel Process Model (EPPM), people’s cognitive appraisal of health threat is an important factor to motivate people to do something about this health threat. When people are presented with a health risk (e.g., HPV), they initially consider two factors: susceptibility and severity of the threat. Susceptibility is defined as people’s subjective perception of the possibility that they will face this threat (e.g., “I should worry about HPV.”). Severity refers to subjective perceptions about the seriousness of the threat (e.g., “I think HPV infection is a serious condition.). Susceptibility combined with severity generally induces people’s perception of threat. It came to our attention that the most of HPV brochures appeared to emphasize its potential cause for the cervical cancer rather than its route of transmission. Frequently, cancer is referred to as the most feared of all illnesses; also cancer is so common that it actually accounts for one out of every four deaths in the United States. Considering Witte’s EPPM, it is somewhat plausible to heighten the fact about cancer in HPV brochure in order to create a substantial health threat for people. Thus, we proposed the following research question:

RQ1: Among the following three conditions [(a) sexually transmitted route, (b) foodborne route, (c) no indication of transmission route, but its possible development for cancer], will the participants in the “cancer” condition perceive higher health threat in terms of the perceived susceptibility and severity than the participants in the other two conditions?

### 1.2 STD-related Stigma and Communication Efficacy

Once individuals realize that they are infected with STDs, they fear that they might be rejected by members of the community and more likely to isolate themselves from society. The anticipated shame or fear after being found out by the community lead the stigma-carrying individuals to engage in secrecy (Markowitz, 1998). According to Newton and McCabe (2005), having a STD is somewhat a unique stigmatizing condition, since the infected individual’s stigmatized symptoms are usually concealable to most people. Thus, stigma-carrying individuals are able to keep their health condition a secret. Keeping these kinds of secrets, however, is believed to bear a high cognitive burden and eventually leads to stress (Pachankis, 2007).

STD-infected women worried about telling their family members because of the stigma of a STD and its association with sexual promiscuity. The qualitative study conducted by McCaffey, Waller, Nazroo, and Wardle (2006) found that STD-infected individuals in the Great Britain reported their family and friends actually being supportive and helpful once they told them about their STD infections; however, a few described negative experiences, such as being judged or blamed by family or friends.

Oftentimes, secrecy is closely related to communication efficacy. Stigma-carrying individuals might not want to reveal the information, but even though they want to reveal it to their close family members or friends, they simply cannot do it because they have low communication efficacy. Communication efficacy concerns individuals’ perceptions that they can successfully engage in the communication in order to gather information (Afifi & Morse, 2009) or, in this context, to minimize the burden coming from secrecy. In the context of health communication, it has been reported that communication efficacy is known as a strong predictor for individual’s communication decisions.

Considering the fact that high perceived stigma (such as STDs) are often associated with secrecy (Cunningham et al., 2002), it is plausible to argue that individuals with STDs might have lower communication efficacy compared to individuals with other non- (or less) stigmatizing diseases. If stigma-carrying individuals decide not to reveal their diseases, even to close family or friends, it would be important to identify the existing factors that prevent them from engaging in communication. To this end, the second hypothesis and a research question were proposed as follows:

H2: Among the following three conditions [(a) sexually-transmitted, (b) foodborne, (c) no indication of transmission route, but its possible development for cancer], the sexually transmitted route will have the lowest score for communication efficacy.

RQ2: What are the reasons that prevent people from engaging in communication about the disease, and do these reasons vary in different conditions?

The current research was also designed to incorporate the impact of the attributions of responsibility with communication outcomes. Weiner’s (1995) decision-stage model holds that behavior is determined by a cognitive process: individuals make attributions about the cause and its controllability of a person’s illness, which lead to inferences about responsibility. These inferences lead to blame, which affects the likelihood of helping or punishing behaviors. In sum, the sequence of judgments is: causal controllability - responsibility - blame - behavioral response. The current study explicated this sequence by substituting behavior responses with a communication variable to examine how attributions of responsibility sequence have an impact on individuals’ communication efficacy in terms of disclosing the disease. Applying Weiner’s (1995) model in this study, we hypothesized that people who are attributed with control over contracting the disease are likely to be assigned greater levels of personal responsibility and would be more likely to receive blame from others. This process will have a significant impact on one’s communication efficacy. In other words, people who are infected with the hypothetical virus (i.e., *SV-41*) by sexual intercourse would be perceived to have had greater controllability of the situation, thus, be assigned more responsibility for the disease. This would lead to greater blame and, ultimately, to lower communication efficacy. The last hypothesis (H3) would test this sequence by proposing a path model.

## 2. Method

### 2.1 Overview of Design

In the present research, the main purposes were to assess (1) individuals’ general attitudes on stigma and perceptions on attribution of responsibility (controllability, responsibility, and blame) toward STDs and (2) the relationship between attribution of responsibility and individuals’ communication efficacy. The basic design for this study involved randomly presenting participants with one of three vignettes (sexually transmitted route vs. foodborne route vs. cancer development) regarding hypothetical virus infection and then asking them to respond to questions about the scenario.

### 2.2 Participants and Procedure

Two hundred and ninety-eight students enrolled in an entry-level communication course at a Midwestern university earned course credits for participating in this online survey study. The participants were 82 males (27%) and 216 females (73%). Participants ranged in age from 18 years to 54 years (*M* = 23.79, *SD* = 5.60). More than half (65.8%) of all participants were Caucasians, 24.0% were African Americans, and 4.3% were Asians. Analyses revealed no significant differences across experimental groups on any of the pretest or demographic variables.

Before collecting data, the study protocol was approved by the IRB. All subjects who wished to participate read the IRB-approved consent form before proceeding to the online survey experiment. Once participants had given their consent, they were asked to answer some demographic questions. Then, participants were randomly placed into one of the three hypothetical situations. After the participants were exposed to the hypothetical scenario, the instructions asked participants to enter their responses to the questionnaires via the computers. After participants completed the online survey questionnaires, they were debriefed and dismissed.

### 2.3 Scenarios

To test the prediction that STDs are implied with more stigma, regardless of their symptoms/conditions, a scenario describing a hypothetical virus, the *SV-41* was developed. Participants were randomly assigned to one of three scenarios, including that the *SV-41* is transmitted through (1) sexual intercourse or (2) food. The third scenario did not indicate the route of transmission, but it described a situation of being infected with (3) a virus that can develop into prostate cancer. This study used and modified a scenario which was originally created by Mantler and her colleagues (2003). The reason why we did not use an actual virus, such as HPV or influenza, was to control for participants’ pre-existing attitudes/perceptions or knowledge associated with the actual virus. In addition, it was easier for us to manipulate the symptoms/conditions for the hypothetical virus infection rather than using an actual virus infection. The scenario for virus infection contracted through sexual intercourse was as follows:

*William is 22 years old, a typical college kid at a Midwestern university (where participants were attending to). He has lots of friends and everyone seems to like him. William is a pretty healthy individual who exercises regularly and tries not to eat much fast food. However, about a week ago, William began to feel a burning sensation when urinating. William also had intestinal symptoms, such as diarrhea and nausea. He decided to see his physician, Dr. Smith. After Dr. Smith ran some tests (i.e., a blood test, a urine test, etc.), William was told that he had tested positive for newly identified virus named “SV-41”. Dr. Smith told William that the SV-41 infection is known to be transmitted by sexual intercourse. Dr. Smith also mentioned that SV-41 is not a deadly virus, but there is no “cure” yet*.

The other scenarios had exactly the same information, but the route of transmission was either different (i.e., “Dr. Smith told William that *SV-41* infection is known to be transmitted by eating food containing this virus.”) or omitted but stating it can be a potential cause of cancer (i.e., “Dr. Smith told William that *SV-41* infection can develop into prostate cancer.”). There were 95 participants in the “sexually transmitted route” condition, 95 participants in the “cancer” condition, and 108 participants in the “foodborne route” condition.

Although the scenarios were using a male as a main character (i.e., William and prostate cancer), no sex difference was found in the perceived susceptibility, *t* (296) = .77, *p* = .44, and in the perceived severity, *t* (296) = .46, *p* = .65. Therefore, female participants as well as male participants perceived *SV-41* to be equally susceptible and severe.

### 2.4 Measures

#### 2.4.1 Attribution Variables

In order to measure the attribution variables, the current study adopted a scale designed by Mantler et al. (2003). Controllability, responsibility, and blame were each measured with scales consisting of four items (two positively and two negatively worded items), using a 5-point Likert-type response format. The controllability dimension focused on whether William had control over a situation, and its Cronbach’s coefficient alpha was .70. The responsibility dimension (Cronbach’s α = .80) attended to whether William was responsible for contracting the infection. The blame dimension (Cronbach’s α = .81 after deleting one item) was assessing if William deserved punishment due to this infection.

#### 2.4.2 Stigma

The perceived stigma was measured by using Fortenberry et al.’s (2002) stigma scale. Their scale was originally developed to measure STD-related stigma, which reflected the participant’s expectation of isolation and adverse social outcome associated with STD, however, it was adapted to fit the purposes of the current study. This measure has six items using a 5-point Likert-type response format. The reported Cronbach’s alpha was .89.

#### 2.4.3 Shame

Items for perceived shame were developed for the current study. Participants responded on a 5-point Likert scale. It was originally a four item scale (α = .79), but one item was deleted in order to have a higher internal consistency (α = .92).

#### 2.4.4 Health Threat

Participants were asked four items, each concerning perceived susceptibility and severity on a 5-point Likert-type response format. The reported Cronbach’s alpha for the perceived susceptibility dimension was .80, and for the perceived severity was .85.

#### 2.4.5 Communication Efficacy

Communication efficacy was measured by asking about their ability to tell other people if participants had the *SV-41* infection themselves. Participants were expected to answer whether they could communicate about their *SV-41* infection with four different targets (family, close friends, girlfriends/boyfriends, and roommates), in a yes/no format. As a filtered question, the reason was asked if they could not talk about *SV-41* to a certain target person. If the participants currently did not have a partner or a roommate, the data was left as missing data. The score was added after data collection, and higher scores indicated more efficacy to talk about *SV-41*.

### 2.5 Intercoder reliability

In order to assess the reason for not communicating about their hypothetical virus, the responses were content analyzed by the authors. Coders evaluated every response. Using Cohen’s kappa (κ) formula, the intercoder reliability was .92. After calculating the intercoder reliability, the few disagreements were resolved by follow-up discussion.

## 3. Results

The first hypothesis predicted that participants would perceive a hypothetical person who was infected with virus in a sexually transmitted route as having the highest levels of the perceived (1) controllability, (2) responsibility, (3) blame, (4) stigma, and (5) shame. Due to the conceptually-related nature of the dependent variables, a one-way MANOVA was conducted to determine the effect of different transmission routes on participants’ general perceptions on stigma/shame and its attributions of responsibility. The MANOVA results indicated that the route of transmission of *SV-41* significantly affected the combined DVs, Wilks’ Λ .59, *F* (10, 582) = 17.32, *p* < .001, partial *η^2^* = .23.

The data were further analyzed separately for the various dimensions of responsibility attributions. A univariate ANOVA was conducted as a follow-up test, and the results indicated that different transmission routes had a significant effect on controllability of the situation, *F* (2, 295) = 71.83, *p* < .001, partial *η^2^* = .33. As expected, when controlling for the symptom/condition of the disease, the participants perceived a person who was infected through a sexually transmitted route as having significantly more control over the situation (*M* = 3.58, *SD* = .86) than via foodborne (*M* = 2.33, *SD* = .71) or no specification of the route but a possibility to develop into cancer (*M* = 2.60, *SD* = .74). Interestingly, the mean difference between a foodborne and a possible cancer development condition was significant (*M*_D_ = .27, *p* < .05) that participants viewed that an infected person with a possible cancer development actually had more control over the situation.

Regarding perceived responsibility, a similar pattern was found in that route of transmission had a significant effect, *F* (2, 295) = 69.13, *p* < .001, partial *η^2^* = .32. The participants held significantly more responsibility to an infected person in the sexually transmitted route scenario (*M* = 3.58, *SD* = .78) than in the foodborne route scenario (*M* = 2.26, *SD* = .87) or cancer scenario (*M* = 2.49, *SD* = .86). The mean difference between a foodborne and a cancer scenario was close to statistical significance, (*M*_D_ = .23, *p* = .053), but, again, participants held more responsibility for the infected person in a cancer development scenario than in a foodborne scenario.

As predicted, the significant main effect of transmission route was found in ratings for blame *F* (2, 295) = 49.87, *p* < .001, partial *η^2^* = .25. Participants who were in the sexually transmitted route scenario (*M* = 3.05, *SD* = .84) blamed the infected person significantly more than participants either in the foodborne route scenario (*M* = 1.89, *SD* = .80) or in the cancer development scenario (*M* = 2.14, *SD* = .93). Again, the infected person in the cancer development scenario received significantly more blame than in a food borne scenario (*M*_D_ = .25, *p* < .05).

Statistical significance was found in the perceived stigma, *F* (2, 295) = 3.60, *p* < .05, partial *η^2^* = .02, and as predicted, participants in the sexually transmitted route scenario perceived the infected person to have significantly higher stigma; however, a slightly different pattern was found in the perceived stigma in that no statistical difference was found between a sexually transmitted route (*M* = 2.91, *SD* = .88) and a foodborne route (*M* = 2.73, *SD* = .92). In addition, the difference between the foodborne route and cancer development (*M* = 2.55, *SD* = .99) was not significant. The only statistical significance found was between a sexually transmitted route scenario and a possible cancer development scenario (*M*_D_ = .36, *p* < .01), which indicated that participants, indeed, associated significantly more stigma with the STD than with cancer.

Lastly, there was a statistical significance in terms of the perceived shame, *F* (2, 295) = 23.22, *p* < .01, partial*η^2^* = .14. The mean pattern was in a predicted direction that participants perceived the highest shame in a sexually transmitted route scenario (*M* = 2.46, *SD* = 1.12), followed by a cancer scenario (*M* = 1.86, *SD* = 1.02, *M*_D_ = .60, *p* < .01), and a cancer development scenario (*M* = 1.52, *SD* = .81, *M*_D_ = .34, *p* < .05), and the mean differences among these three conditions were all significant.

Research question 1 was proposed to assess the health threat, more specifically, whether the word “cancer” actually increased participants’ perceived susceptibility and severity of the disease. A one-way MANOVA result indicated the different condition had a significant impact on the perceived susceptibility and severity as a whole, Wilks’ Λ .93, *F* (4, 588) = 5.68, *p* < .001, partial *η^2^* = .04. A further univariate ANOVA result indicated that statistical significance was found in the perceived susceptibility, *F* (2, 295) = 3.62, *p* < .05, partial *η^2^* = .02, but it was mainly due to participants in a foodborne scenario who perceived themselves to be significantly more susceptible to the condition (*M* = 3.00, *SD* = .95) than participants either in a cancer scenario (*M* = 2.73, *SD* = 1.04, *M*_D_ = .27, *p* = .051) or in a sexually transmitted route scenario (*M* = 2.65, *SD* = .95, *M*_D_ = .35, *p* < .05). In terms of the perceived severity, a univariate ANOVA revealed the statistical significance at *p*-level of .10, *F* (2, 295) = 2.19, *p* = .06, partial *η^2^* = .02; however, a somewhat different result was found in that participants perceived the sexually transmitted route (*M* = 3.27, *SD* = .93) as having significantly higher severity than the foodborne route (*M* = 2.99, *SD* = .82, *M*_D_ = .29, *p* < .05), but the difference between the sexually transmitted route scenario and the cancer development scenario (*M* = 3.18, *SD* = .87, *M*_D_ = .09) was not significant. This result indicated that the word “cancer” was not powerful enough to increase the perceived health threat.

Hypothesis 2 predicted that the route of transmission would have a significant impact on participants’ communication efficacy. As predicted, hypothesis 2 received support, *F* (2, 165) = 4.65, *p* < .05, in that participants in a foodborne route condition indicated that they could communicate about *SV-41* infection with significantly more number of people (*M* = 3.91, *SD* = .34) than participants either in a cancer development condition (*M* = 3.61, *SD* = .76, *M*_D_ = .30, *p* < .05) or in a sexually transmitted route (*M* = 3.60, *SD* = .77, *M*_D_ = .31, *p* < .05); however, the mean difference between the cancer development condition and the sexually transmitted condition was not statistically significant, which indicated that participants in both conditions had similar levels of communication efficacy.

More specifically, 42% (40 of 95) of all participants in the sexually transmitted route scenario and 36% (34 of 95) of all participants in the cancer scenario felt that they could not (or did not want to) tell other people about their virus infection. Only 11% (12 of 108) of all participants in the foodborne scenario, however, felt that they could not engage in conversation about their *SV-41* infection.

As a follow-up question, research question 2 asked about the reasons that would prevent people from engaging in communication about the hypothetical virus infection and if these reasons varied in different conditions. In sexually transmission, 32.5% of the participants indicated that their lack of communication efficacy was rooted in fear of being stigmatized (i.e., being judged by people or being ashamed). Some of the participants described, “I think that they would think less of me. They would also feel that I let them down by being irresponsible” or “Because it is sexually transmitted, my family would look down upon me.” 20% of the participants indicated that it was too private to discuss with other people (including their family).

In the cancer development scenario, among those of who decided not to tell other people about their virus infection, 23% of the participants stated that they were afraid of being stigmatized by others, and 32% of the participants stated that it was too private to discuss with other people. A few participants indicated that they were not close enough to talk about the infection with their family or roommates. Three participants (8%) indicated that they were not willing to engage in communication about the infection because they did not want others to be worried about them. A few participants wrote, “The reason I would not tell them is because I would not want them to be all worried about me” or “I wouldn’t want them to worry about a condition that has nothing to do with them. It’s one less thing for them to have going in their busy schedule.”

Compared to the other two conditions, a quite different result was found in the foodborne scenario in that significantly less participants (8%, 1 of 12) worried about being stigmatized from the virus infection. 33% (4 of 12) of the participants would not communicate because they did not want others to worry about the infection. The underlying reason was slightly different from the cancer scenario, however, in that participants did not want others to worry about the infection being contagious.

**Figure 1:**
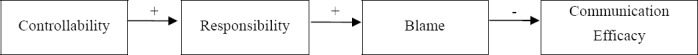
Hypothesized model

**Figure 2:**
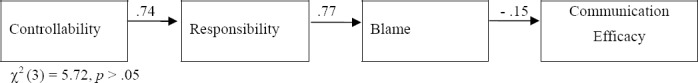
Hypothesized model with path coefficients

In order to further examine these data for a mediating relationship between attributions of responsibility sequence and communication efficacy, a path model was proposed (H3). In order to test the proposed model (see Figure 1), the ordinary least squares criterion (Hunter & Gerbing, 1982) was employed to estimate the parameters, examine the parameter size, and finally evaluate the fit of the model. The zero-order correlations in Table 1 were employed to estimate the model parameters, and the path coefficients are given in Figure 2.

**Table 1:** Zero-order correlation matrix used to calculate parameter estimates

	1	2	3	4
				
Controllability	-			
Responsibility	.74**	-		
Blame	.69**	.77**	-	
Communication Efficacy	-.16**	-.15**	-.15**	-

** Significant at p < .01 (2-tailed)

Parameter size was estimated for the path diagram by performing a simple regression of each endogenous variable onto its causal antecedent (See Hunter & Gerbing, 1982 for information on reproducing correlations in path analysis). Then, a model fit was performed by comparing the estimated parameter size to the reproduced correlations. In order to say that the model is consistent with the data, two conditions should be met: (1) the path coefficients must be substantial and (2) the discrepancies between the parameter estimates and the reproduced correlations must be attributable to sampling error. If the errors were larger than what was expected from sampling error, the model would be regarded as inconsistent with the data.

From Figure 2, it can be concluded that each of the path coefficients is both substantial and in the predicted direction. As predicted by Weiner’s decision-stage model (1995), the coefficient linking controllability and responsibility was .74, *P* (.69 ≤ ρ ≤ .79) = .95, indicating that controllability had a significant effect on responsibility. Responsibility, in turn, affected blame (path coefficient = .77, *P* (.73 ≤ ρ ≤ .82) = .95), such that the more responsibility an infected person is said to hold, the more blame they deserve to receive. Lastly, blame predicted communication efficacy (path coefficient = -.15, *P* (.04 ≤ ρ ≤ .27) = .95), such that if individuals perceive more blame, their communication efficacy is decreased.

The discrepancies between predicted and obtained correlations for all unconstrained bivariate relationships in the model were also examined, and none differed substantially from what was expected from sampling error. In addition, using Baron and Kenny’s (1986) mediational analysis, the mediated relationships were tested. The results indicated that the relationship between controllability and blame (r = .74) dropped substantially to r = .28, *p* < .05 when controlling for responsibility; the relationship between responsibility and communication efficacy (r = - .15) became r = - .06, *n.s* when controlling for blame.

The global test for goodness of fit indicated that the data were consistent with the model, χ^2^ (3) = 5.72, p > .05. Given that the path coefficients were relatively large and that the model and parameter estimates accurately predicted the unconstrained correlations, the model and data were judged to be consistent with one another.

## 4. Discussion

The current study explored the mechanism of how individuals formed stigma associated diseases have different transmission routes and how the attributions of responsibility process had an impact on communication efficacy. As predicted, people attributed with greater control over contracting the virus (i.e., sexually transmitted route) were likely to be assigned a greater level of personal responsibility and would be more likely to receive blame from others than people attributed with relatively less control over contracting the virus (i.e., foodborne). This process of the attributions of responsibility had a significant impact on communication efficacy, in that the perceived controllability of the situation, perceived responsibility for the situation, and blame were all significantly correlated with communication efficacy in a negative direction.

In order to control for preexisting attitude or knowledge associated with actual diseases, this study created a hypothetical virus, *SV-41*. Also, by using a hypothetical virus, we could control for the symptoms/conditions for the disease. Interestingly, although the symptoms/conditions were controlled across the experimental conditions, and only the route of transmission was manipulated, the perceptions of the virus infection, in terms of stigma, were significantly different. It was interesting to observe, that in spite of having vague information regarding how the target person was infected with *SV-41*, participants simply regarded being infected with a sexually transmitted virus as more stigmatizing and shameful than being infected with a foodborne virus. Participants seemed to assume that being infected via the foodborne route was a consequence of “passive” and “normal” behavior that the target person had no or relatively less control over the situation, while being infected via sexual intercourse was a consequence of “active” and “non-normative” behavior, and that the target person could have made a different decision to not be infected.

This investigation also aimed at assessing (1) the effect of hiding a stigmatizing phrase, “sexually transmitted virus,” on shaping individuals’ perceived stigma and shame and (2) the effect of presenting the word “cancer” on shaping individuals’ perceived health threat. In other words, this study examined if drug company Merck’s HPV advertisement strategies were effective in terms of minimizing the stigma associated with the sexually transmitted nature of HPV and idealizing the level of health threat, which was measured by perceived severity and susceptibility. When participants did not know the transmission route of the hypothetical virus but knew that this virus infection was related to potential prostate cancer development, their perceived shame and stigma were significantly lower than when they knew it was a sexually transmitted virus. Out of curiosity, the correlations among the perceived shame, stigma, and communication efficacy were assessed. Although stigma was not significantly correlated with communication efficacy (*r* = - .05, *n.s*), a significant correlation coefficient was found between shame and communication efficacy (*r* = - .13, *p <* .05). This finding has a practical implication in that Merck’s strategic plan for Gardasil appears to be successful in terms of reducing the perceived shame and stigma associated with the sexually transmitted nature of the virus. By not revealing the true nature of the virus upfront, they may prevent people from experiencing additional anxiety and distress from stigmatization (McCaffery et al., 2006).

The effect of the word “cancer” was not powerful enough to increase one’s health threat, which was assessed by the perceived severity and susceptibility. Participants felt a similar magnitude of perceived susceptibility between a possible cancer development scenario and a sexually transmitted route scenario. In terms of the perceived severity, though no statistical significance was found, the sexually transmitted route appeared to be perceived to be the most severe of all conditions.

Although the current study did not find a significant impact of using the word “cancer” on increasing people’s health threat, in reality, cancer still seems to be the most feared of all illnesses. According to the report for the General Public Focus Group in STD Communication Database (“U.S Department of Health and Human Services,” 2004), anxiety and tension among focus group participants were observed when the relationship between HPV and cervical cancer was introduced. Furthermore, among those women who have received abnormal cervical smear results indicated that they experienced anxiety and fears about cancer as leading psychological sources of distress (Anhang et al., 2004).

We speculated that the main reason why this finding was not significant might be found in the study design, which was an online survey. Also, the survey questionnaire indicated upfront that this was a hypothetical situation; consequently, the design might lack experimental realism. Therefore, for a future study, the issue of ecological validity should be explicitly readdressed by utilizing an existing virus (i.e., HPV). It would be interesting to see if participants’ perceptions of susceptibility/severity differed if they were informed or not informed about how HPV is a virus that can develop cervical cancer. Moreover, it will be meaningful to study the relationship between participants’ perceived the health threat when they learn that HPV can cause cancer and their motivation to engage in appropriate actions. Witte’s (1994) EPPM can be applied in this context in that, when participants perceive a health threat by being exposed to the fact that HPV might cause cervical cancer, they will either attempt to control the danger (e.g., take a positive/active action toward the threat) or their fear (e.g., avoid the issue). It will be theoretically and practically worthwhile to investigate a sufficient level of health threat in the context of HPV.

In addition, our study suggests areas of future investigation by advancing a possible extension of Witte’s EPPM. The EPPM has two parts: threat and efficacy. The first part, *threat*, is a cognitive appraisal that motivates action, *efficacy*. The threat part of the model is usually measured by the perceived susceptibility and severity. The EPPM indicates that, as people feel more threatened based on the perceived susceptibility and severity, they have greater motivation to engage in behaviors that can reduce the cause of the threat. We would like to add one more component to the threat part, the perceived stigma of the disease. If perceived susceptibility and severity are related to one’s physical threats, then it can be speculated that the perceived stigma can induce one’s psychological threat. It also resonates with Smith and her colleagues’ argument that fear of being stigmatized can also motivate people to act, but if their fear is greater than their ability to deal with the stigma, people are more likely to engage in behaviors that control their fear (Smith, Ferrara, & Witte, 2007). Because of this, future studies should examine, when controlling for the perceived susceptibility and severity, if the perceived stigma as a source of threat has a significant impact on shaping one’s self-efficacy. In this context, self-efficacy does not only concern the behavioral capacity of how effectively an individual can perform the recommended responses but also concerns the psychological capacity which encompasses individuals’ perception of their ability to deal with emotional burdens that come from being stigmatized by other people and carry out an action with persistence in spite of facing challenges and barriers (Smith et al., 2007). Smith and her colleagues (2007) also noted that there is a lack of empirical evidence of how sociocultural factors surrounding threat have an impact on one’s efficacy formation.

If the perceived stigma has a negative influence on forming self-efficacy, it should be the next step to investigate the way to minimize the perceived stigma associated with various diseases. The current study did not assess the effect of prevalence and its “normalizing” function and left room for future studies to come in. Waller et al. (2007) found that women who were aware of the high prevalence of HPV infection experienced significantly lower level of stigma, shame, and anxiety than women who underestimated the prevalence of infection. Specifically, women who were presented with prevalence information (e.g., “It is thought that around 70% of people will be infected with HPV at some point during their lives – it is very common.”) did not significantly increase their sense of stigma and anxiety, although they were also aware of the sexually transmitted nature of HPV infection. This “normalizing” effect could be the key factor in mitigating the perceived stigma and negative emotions associated with highly stigmatizing diseases, such as STDs.

The current study, as an exploratory step, introduced a practical implication for how to tailor health messages in order to maximize the awareness of the health threat among people but at the same time to mitigate the perceived stigma and negative emotional outcomes associated with a certain diseases. Stigma is known as the leading barrier to health promotion, treatment, and support (Smith, 2007a). In addition, regardless of transmission route, patients with a stigmatized illness may suffer from receiving less funding for finding treatments or cures or even less adequate care (Lee, Chiu, Tsang, Chui, & Kleinman, 2006). Therefore, in public campaigns, more effort should be made to reduce stigma towards a certain diseases by making strategic plans to change one’s judgment of attributions toward these diseases.
